# Chronic Kidney Disease Increases Risk of Incident HFrEF Following Percutaneous Coronary Intervention

**DOI:** 10.3389/fcvm.2022.856602

**Published:** 2022-04-01

**Authors:** Wenguang Lai, Xiaoli Zhao, Sijia Yu, Ziling Mai, Yang Zhou, Zhidong Huang, Qiang Li, Haozhang Huang, Huanqiang Li, Haiyan Wei, Dachuan Guo, Yun Xie, Shanggang Li, Hongyu Lu, Jin Liu, Shiqun Chen, Yong Liu

**Affiliations:** ^1^School of Biology and Biological Engineering, South China University of Technology, Guangzhou, China; ^2^Department of Cardiology, Guangdong Cardiovascular Institute, Guangdong Provincial People's Hospital, Guangdong Academy of Medical Sciences, Guangzhou, China; ^3^Department of Guangdong Provincial Key Laboratory of Coronary Heart Disease Prevention, Guangdong Cardiovascular Institute, Guangdong Provincial People's Hospital, Guangdong Academy of Medical Sciences, Guangzho, China; ^4^Department of Cardiology, The Third Affiliated Hospital of Sun Yat-sen University, Guangzhou, China; ^5^The Second School of Clinical Medicine, Southern Medical University, Guangzhou, China; ^6^Department of Cardiology, The First People's Hospital of Kashgar Prefecture, Kashgar, China; ^7^Department of Cardiology, Sun Yat-sen Memorial Hospital, Sun Yat-sen University, Guangzhou, China

**Keywords:** chronic kidney disease, percutaneous coronary intervention, coronary artery disease, heart failure with reduced ejection fraction, left ventricular ejection fraction, incidence

## Abstract

**Background:**

Chronic kidney disease (CKD) is very common in patients who are at a high risk of developing incident heart failure with reduced ejection fraction (HFrEF). However, the harmful effect of CKD on incident HFrEF has not yet been examined among patients with coronary artery disease (CAD) undergoing percutaneous coronary intervention (PCI).

**Methods:**

Patients undergoing PCI with baseline left ventricular ejection fraction (LVEF) ≥ 40% were included from January 2007 to December 2018 (ClinicalTrials.gov NCT04407936). We defined incident HFrEF as a follow-up LVEF of <40% within 3–12 months after discharge. Multivariable logistical regression was performed to examine the association of CKD with incident HFrEF.

**Results:**

Overall, of 2,356 patients (mean age 62.4 ± 10.7 years, 22.2% women), 435 (18.5%) had CKD, and 83 (3.5%) developed incident HFrEF following PCI. The rate of incident HFrEF in the CKD group was higher than that in the non-CKD group (6.9 vs. 2.8%; *p* < 0.001). Multivariate logistic regression analysis indicated that CKD was an independent risk factor of incident HFrEF [adjusted odds ratio (aOR) = 1.75; 95% CI, 1.03–2.92; *p* = 0.035] after adjustment for confounders including age, gender, diabetes, hypertension, atrial fibrillation, congestive heart failure (CHF), baseline LVEF, ACEI/ARB, and statins. Furthermore, patients with incident HFrEF have a higher ratio of all-cause mortality compared to those without HFrEF (26.5 vs. 8.1%; *p* < 0.001).

**Conclusions:**

Our results suggested that CKD was associated with increased risk of incident HFrEF, which was related to higher all-cause mortality in patients with CAD undergoing PCI. On this basis, more aggressive measures should be taken to prevent patients with CKD undergoing PCI from developing HFrEF.

## Introduction

Coronary artery disease (CAD) is the most common cause of heart failure (HF) (1). Even after percutaneous coronary intervention (PCI), patients with CAD still have a high risk of incident heart failure with reduced ejection fraction (HFrEF) (2). Thus, it is necessary to identify risk factors and improve prognosis among patients with CAD undergoing PCI.

Chronic kidney disease (CKD) has been recognized as a leading public health problem worldwide, with a global estimated prevalence of up to 13.4% (3). Previous studies suggested that patients with CKD constitute an increasing proportion of PCI population (4). Meanwhile, patients with CKD are at high risk for adverse cardiovascular events such as recurrent myocardial infarction (MI), HF, and stroke (5–7). Patients with CKD are prone to induced systemic inflammation, volume overload, renin-angiotensin system activation, oxidative stress, and abnormal calcium transport (7–9). These changes also play an essential part in the development of HFrEF (10–13). PCI can improve revascularization, protect the myocardium, and could delay the development of ventricular remodeling and HFrEF in patients with CAD (14). However, whether CKD is independently related to increased risk of incident HFrEF following PCI remains unknown.

Accordingly, we sought to investigate the effect of CKD on incident HFrEF among patients undergoing PCI in a large Chinese population. On this basis, clinicians could be guided to identify early and intervene on incident HFrEF following PCI.

## Methods

### Data Sources and Study Population

**Figure 1:**
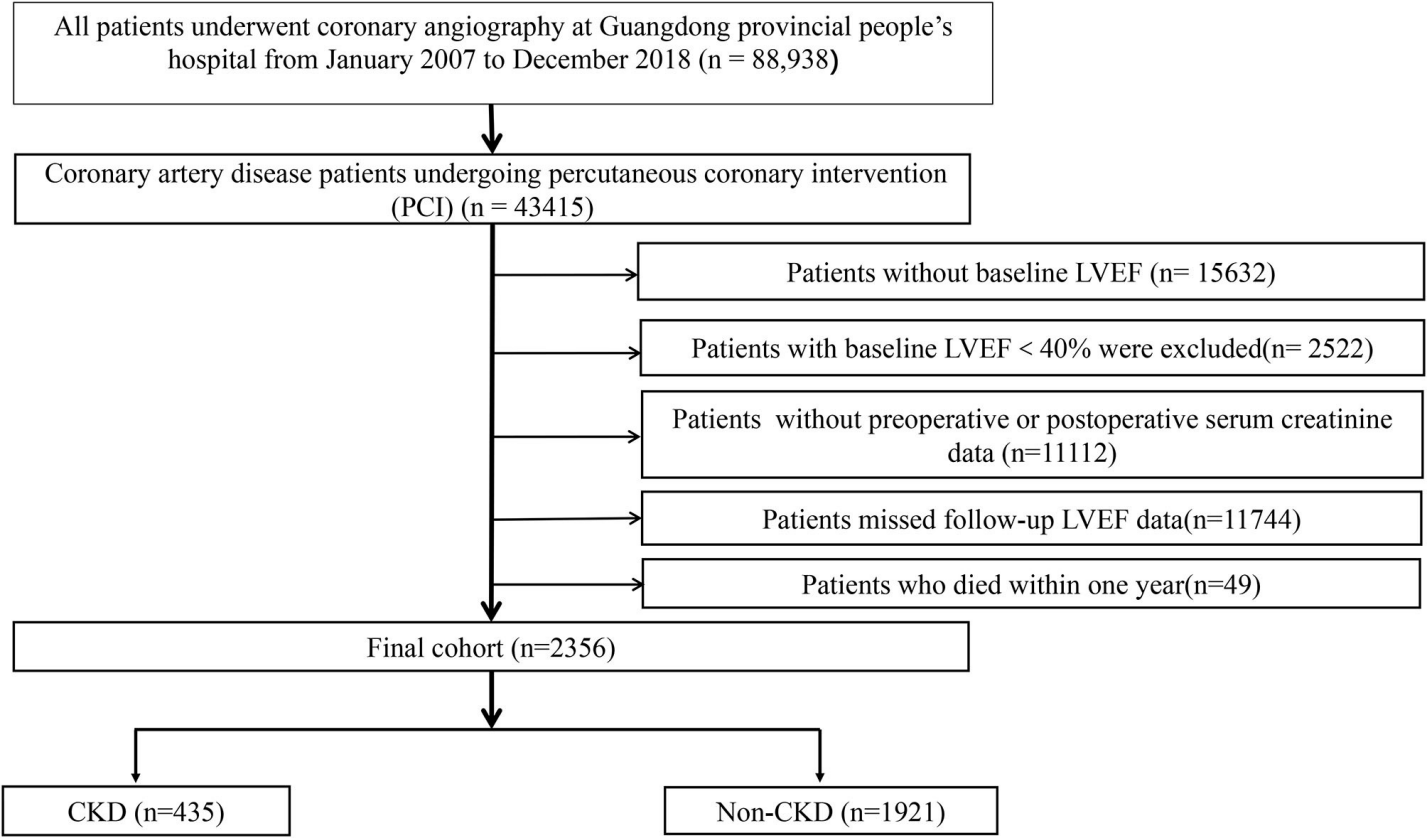
Flow chart of the study population. LVEF, left ventricular ejection fraction; CKD, chronic kidney disease.

We collected baseline information from the registry of Cardiorenal Improvement (CIN) study (ClinicalTrials.gov NCT04407936) from January 2007 to December 2018 at Guangdong Provincial People's Hospital. PCI was performed on patients according to standard clinical practice guidelines (15–17). A total of 2,356 patients with CAD undergoing PCI were included in this research. Exclusion criteria were as follows: (I) lack of baseline left ventricular ejection fraction (LVEF), (II) baseline LVEF <40%, (III) missing 3–12 month follow-up LVEF after discharge, (IV) lack of preoperative or postoperative creatinine data, (V) patients who died within 1 year after discharge (Figure 1). All the patients were classified into the CKD and non-CKD groups. All personal details were erased to protect data confidentiality of the patients. This clinical study was approved by the Guangdong Provincial People's Hospital ethics committee (no. GDREC2019555H) and complied with the declaration of Helsinki.

### Collection of Baseline Data

All data were collected from the standard clinical electronic medical record system of Guangdong Provincial People's Hospital. All medical records can be accessed to ensure that we have baseline information, such as demographic characteristics, medical history, laboratory examination, and medications on discharge. Two experienced cardiac ultrasound doctors performed echocardiography using the biplane Simpson method from apical 4- and 2-chamber views for the whole cohort during hospitalization. All the cardiac ultrasound doctors have undergone unified training, and senior doctors were responsible for quality control of the ultrasound reports. Follow-up LVEF was calculated by with same method above. Median follow-up echocardiography time was 3.5 months, and median follow-up was 4 years.

### Definition of Clinical Outcomes and Variables

The primary outcome was incident HFrEF, defined as a follow-up LVEF of <40% from 3 to 12 months after discharge. HFrEF was defined as LVEF ≤ 40 (18), and CKD was defined as estimated glomerular filtration rate (eGFR) calculated using the Modification of Diet in Renal Disease equation <60 ml/min/1.73 m^2^ (10). Congestive heart failure (CHF) was defined as New York Heart Association (NYHA) class > 2 or Killip class > 1 (19). Diagnoses of acute myocardial infarction (AMI), diabetes mellitus (DM), and hypertension were identified according to ICD-10.

### Statistical Analysis

In this study, the patients were divided into the CKD group and the non-CKD group. Descriptive analysis for continuous variables was shown as means ± standard deviation or medians and interquartile ranges according to distribution. Categorical variables were described as proportions. Kolmogorov-Smirnov test was performed for continuous variables if conforming to normal distribution. Differences in baseline characteristics were compared between the two groups *t*-test for continuous variables, chi-square test for categorical variables, and Kruskal-Wallis test for abnormal distribution. Time-to-event data were shown in graphs using Kaplan-Meier curves. We calculate sample size according to the rule of thumb of Vittinghoff, Peduzzi, and Harrell (20–22). Namely, a number of events per variable (EPV) of 5 or 10 or greater was applied for a multivariate regression model. Multivariate logistic regression was performed to assess the relationship between CKD and incident HFrEF in patients undergoing PCI by expressing it as odds ratio (OR) with 95% confidence interval (CI). The missing values of all adjusted variables in the regression model were <5%, and we computed a model using multiple imputations with chained equations to fill missing data in the adjusted variables. Three types of models were constructed: unadjusted (model 1), adjusted only for age and gender (model 2), and adjusted for age, gender, DM, hypertension, CHF, atrial fibrillation (AF), baseline LVEF, angiotensin-converting enzyme inhibitors/angiotensin receptor blockers (ACEI/ARB), and statins (model 3). All statistical analyses were performed using R (ver. 4.0.3). A 2-tailed *P* value < 0.05 was considered statistically significant.

## Results

### Clinical and Procedural Characteristics

A total of 2,356 patients with CAD undergoing PCI were enrolled, and included 1,834 males and 522 females (mean age 62.7 ± 10.7 years). Among the patients, 1,421 (60.3%) had hypertension, 730 (31%) had DM, 240 (10.2%) had CHF, 634 (26.9%) had AMI, 123 (5.2%) had valvular heart disease, and 62 (2.6%) had atrial fibrillation (AF). The total population was classified into the CKD group (*n* = 435) and the non-CKD group (*n* = 1,921). Patients with CKD were older and more likely to have hypertension, DM, AF, and HF. The CKD group had lower usage of ACEI/ARB, statins, and calcium channel blockers (CCBs). Detailed clinical characteristics of the patients are listed in Table 1.

**Table 1:** Baseline characteristics of patients with and without CKD undergoing PCI.

**Characteristics**	**Overall**	**Non-CKD**	**CKD**	***P*-Value**
	**(*N* = 2,356)**	**(*N* = 1,921)**	**(*N* = 435)**	
**Demographic**				
Age, years	62.4 (10.7)	61.3 (10.6)	67.4 (10.0)	<0.001
Gender, *n* (%)	522 (22.2)	393 (20.5)	129 (29.7)	<0.001
**Medical history and clinical condition**				
AMI, *n* (%)	161 (6.8)	137 (7.1)	24 (5.5)	0.270
AF, *n* (%)	62 (2.6)	39 (2.0)	23 (5.3)	<0.001
VHD, *n* (%)	123 (5.2)	98 (5.1)	25 (5.8)	0.671
HT, *n* (%)	1,412 (60.3)	1,076 (56.04)	345 (79.3)	<0.001
DM, *n* (%)	730 (31.0)	557 (29.0)	173 (39.8)	<0.001
CHF, *n* (%)	240 (10.2)	167 (8.7)	73 (16.8)	<0.001
**Laboratory examination**				
CK-MB (U/L)	10.70 [7.30, 16.10]	10.80 [7.30, 16.52]	10.40 [6.93, 15.20]	0.140
GLU (mmol/L)	7.46 (3.41)	7.32 (3.33)	8.11 (3.69)	0.001
HbA1c (mmol/L)	6.62 (1.40)	6.57 (1.38)	6.83 (1.44)	0.005
LDL-C (mmol/L)	2.91 (1.02)	2.92 (1.04)	2.83 (0.94)	0.113
HDL-C (mmol/L)	0.98 (0.24)	0.99 (0.24)	0.96 (0.24)	0.023
HGB (g/L)	133.28 (17.25)	135.20 (15.62)	124.99 (21.10)	<0.001
eGFR (ml/min/1.73 m^2^)	77.27 (24.69)	86.42 (18.32)	44.30 (14.41)	<0.001
NT-Pro-BNP (pg/ml)	293.80 [78.32, 972.75]	232.80 [63.50, 739.30]	885.70 [258.12, 2,840.00]	<0.001
**Echocardiography**				
LVEF (%)	60.25 (9.1)	60.63 (9.0)	58.59 (9.7)	<0.001
LVEDD (mm)	47.93(5.9)	47.83 (5.8)	48.37 (6.5)	0.085
LVESD (mm)	31.28 (6.6)	31.13 (6.5)	31.94 (7.0)	0.022
MAP (mmHg)	0.83 (0.8)	0.82 (0.8)	0.89 (0.2)	0.066
MEP (mmHg)	0.74 (0.2)	0.74 (0.2)	0.77 (0.3)	0.025
PWLV (mm)	10.02 (1.6)	9.92 (1.5)	10.42 (1.8)	<0.001
VS (mm)	10.62 (1.8)	10.49 (1.8)	11.18 (2.0)	<0.001
**Treatment during hospitalization**				
ACEI/ARB, *n* (%)	1,303 (55.4)	1,092 (56.9)	211 (48.7)	0.002
β-blocker, *n* (%)	2,051 (87.2)	1,674 (87.2)	377 (87.0)	0.969
Statins, *n* (%)	2,318 (98.6)	1,896 (98.9)	442 (97.5)	0.045
CCB, *n* (%)	565 (24.0)	402 (21.0)	101(37.6)	<0.001
**Follow up**				
Follow_up_death, *n* (%)	207 (8.8)	141 (7.3)	66 (15.2)	<0.001
HFrEF, *n* (%)	83 (3.5)	53 (2.8)	30 (6.9)	<0.001

*CKD, chronic kidney disease; AMI, acute myocardial infarction; DM, diabetes mellitus; CKD, chronic kidney disease; CHF, congestive heart failure; AF, atrial fibrillation; VHD, valvular heart disease; PCI, percutaneous coronary intervention; HbA1c, glycosylated hemoglobin; LDL-C, low-density lipoprotein cholesterol; HDL-C, high-density lipoprotein cholesterol; CK-MB, creatine kinase isoenzyme-MB; GLU, glucose; HGB, hemoglobin; eGFR, estimated glomerular filtration rate; NT-Pro-BNP, N-terminal pro-brain natriuretic peptide; LVEF, left ventricular ejection fraction; LVEDD, left ventricular end-diastolic dimension; LVESD, left ventricular end-systolic dimension; MAP, mean artery pressure; MEP, mean effective pressure; PWLV, posterior wall of left ventricle; VS, ventricular septum; ACEI/ARB, angiotensin-converting enzyme inhibitor/angiotensin receptor blocker; CCB, calcium channel blocker. HFrEF, heart failure with reduced ejection fraction*.

We found that patients with incident HFrEF were older and more likely to combine with CHF, and they had a lower level of eGFR and baseline LVEF, but had larger left ventricular end-diastolic dimension (LVEDD) and left ventricular end-systolic dimension (LVESD). Detailed information is provided in Supplementary Table 1.

### Prevalence of Incident HFrEF

**Figure 2:**
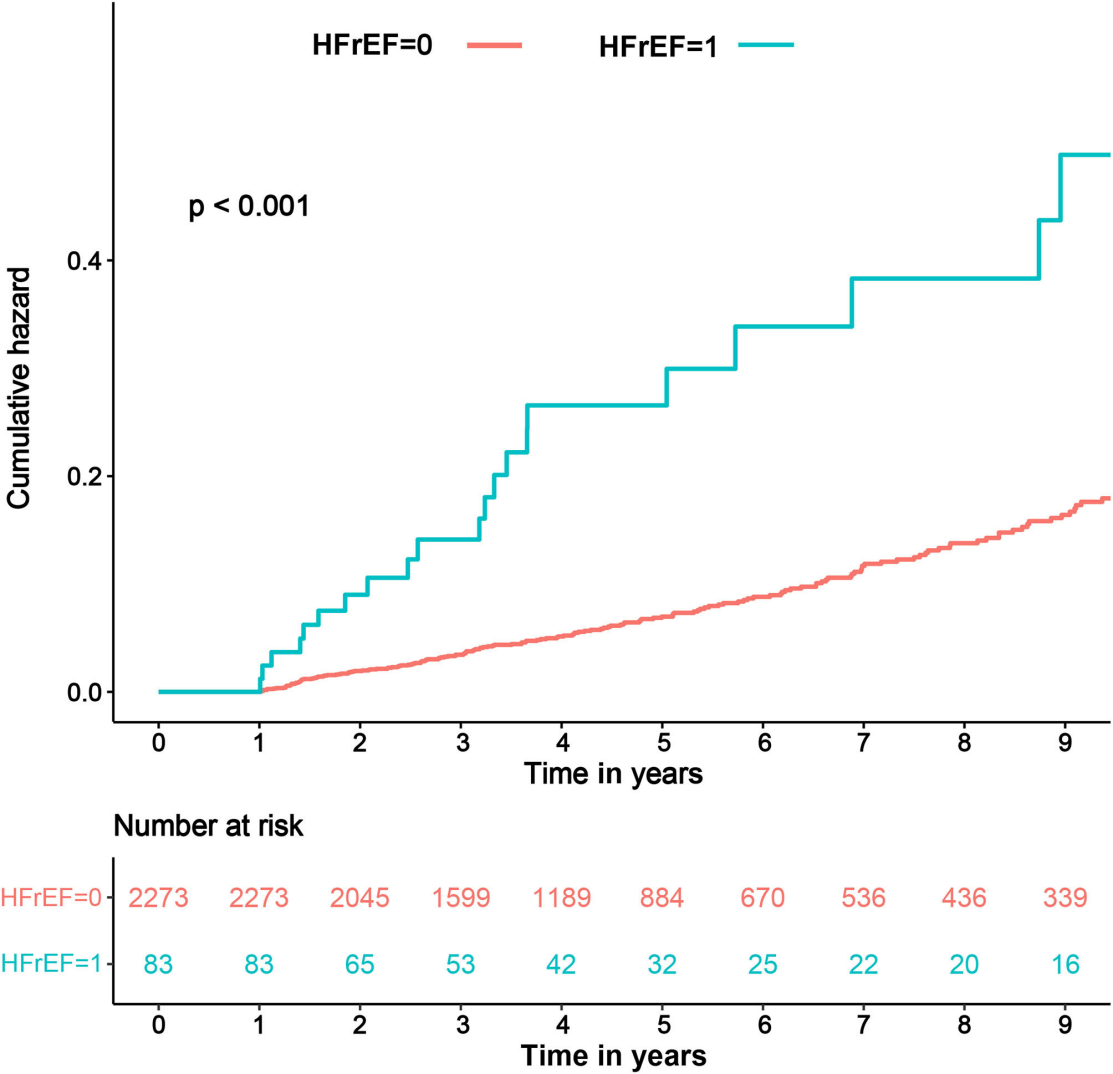
Kaplan-Meier curve in terms of all-cause mortality in patients with and without HFrEF. HFrEF, heart failure with reduced ejection fraction; HFrEF = 1, patients with incident HFrEF; HFrEF = 0, patients without incident HFrEF.

Echocardiographic data from all the patients during the 3- to 12-month follow-up revealed that there were 83 (3.5%) cases of incident HFrEF. The prevalence of incident HFrEF was 6.9% in the CKD group and was 2.5 times higher than that in the non-CKD group. In addition, Kaplan-Meier curves showed higher all-cause mortality in patients with incident HFrEF than in those without HFrEF (log-rank test, *P* < 0.001) (Figure 2).

### Association Between Chronic Kidney Disease and Incident HFrEF

**Figure 3:**
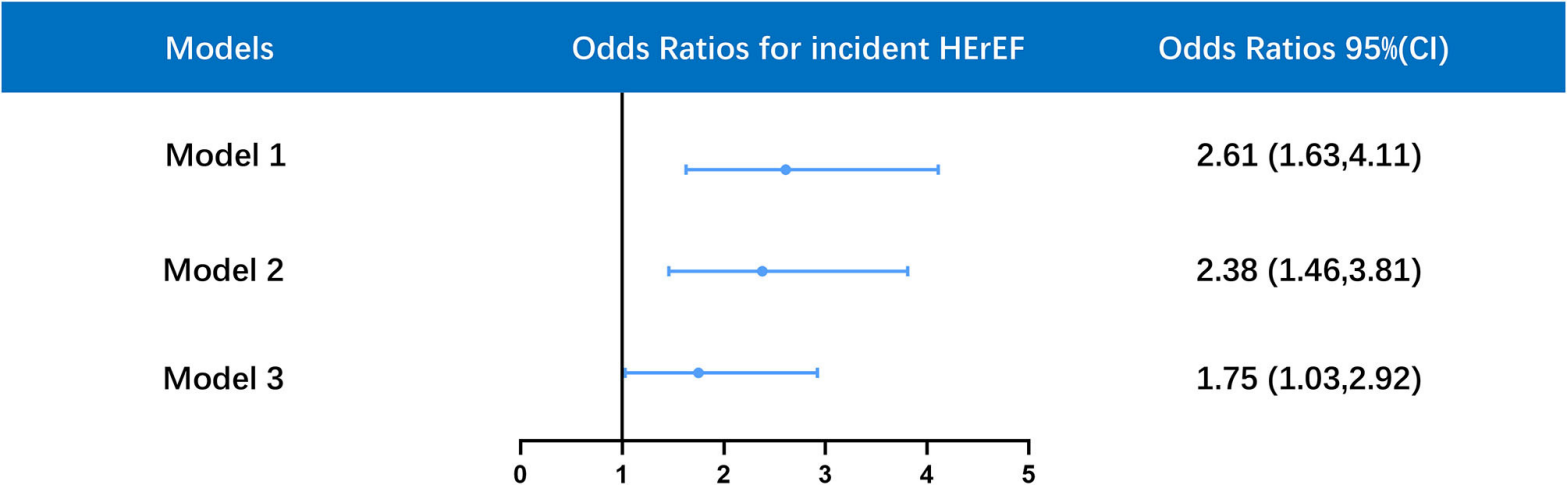
Multivariate logistic regression analysis for association between HFrEF and CKD in different models. Model 1 was unadjusted; model 2 was only adjusted for age and gender; model 3 was adjusted for age, gender, diabetes mellitus (DM), hypertension, atrial fibrillation (AF), congestive heart failure (CHF), baseline left ventricular ejection fraction (LVEF), angiotensin-converting enzyme inhibitor/angiotensin receptor blocker (ACEI/ARB), statins. OR, odds ratios; CI, confidence interval. (ROC: AUC = 0.844, Hosmer-Lemeshow goodness-of-fit test: *P* = 0.11).

Univariate logistic regression analysis revealed that CKD was positively related to incident HFrEF [OR 2.61, 95% CI 1.63–4.11, *p* < 0.001]. There was also a significant association between CKD and incident HFrEF after adjustment for age and gender [OR 2.38, 95% CI 1.46–3.81, *p* < 0.001]. This association remained significant even after further adjustment for age, gender, diabetes, CHF, AF, hypertension, ACEI/ARB, statins, and baseline LVEF [OR 1.75, 95% CI 1.03–2.92, *p* = 0.035] (Figure 3).

## Discussion

To our knowledge, this is the first study to analyze the relationship between CKD and incident HFrEF following PCI. In our research, patients with CKD had a higher rate of incident HFrEF. The overall prevalence of incident HFrEF was 3.5%, and the incidence of HFrEF was 2.5-fold higher in the CKD group than in the non-CKD group. CKD was an independent risk factor for incident HFrEF, which was associated with higher all-cause mortality. Our findings pointed to the importance of accounting for CKD in assessing the risk of incident HFrEF. Therefore, targeting CKD should be considered as one focus of prevention efforts for incident HFrEF among patients undergoing PCI.

Previous studies have shown that CKD and CAD play a critical role in the progression of HF. Kottgen et al. reported that the incidence of HF was 3-fold higher in the CKD group than the non-CKD group, and that the incidence of HF in those with CAD was up to five times higher than in those without CAD in a cohort of 14,857 adults in the United States (18). PCI can improve revascularization, protect the myocardium, and could delay the occurrence of HFrEF and ventricular remodeling. Gu et al. found that only 4.6% patients had an occurrence of incident HFrEF following PCI during a median follow-up of 63 months in a cohort of 3,910 patients with CAD (2). However, there is still a paucity of data on the risk of incident HFrEF among patients with CKD undergoing PCI. Our results found that the incidence of HFrEF in patients with CKD undergoing PCI was up to 6.9% within 1 year, which indicated that the risk of incident HFrEF remained high even after timely revascularization.

Furthermore, previous research studies have shown that renal insufficiency has been proposed as a risk factor for HF. Kottgen et al. reported that there was a 95% increased risk of developing HF in patients with CKD compared to patients without CKD (18). Additionally, Domingo et al. also found that renal insufficiency was a strong predictor of HF development among 2,763 women with CAD (23). Similarly, our previous study has shown that acute kidney injury could increase the risk of ventricular remodeling in patients undergoing CAG (24), which indicated that there was a close relationship between renal dysfunction and HF. Patients with CAD still have a risk for incident HFrEF even after PCI and optimal drug therapy (2). Accordingly, based on previous research studies, we explored further the effect of CKD on incident HFrEF among patients undergoing PCI. These two studies showed that renal dysfunction was an independent risk factor for deterioration of cardiac function regardless of transient or chronic impairment. Therefore, clinicians need to pay attention to changes in renal function.

Previous studies have often investigated the effect of CKD on left ventricular function for more than 3 years follow-up (18, 23, 25). Metabolic and hormonal derangements, accompanied with CKD, could impair left ventricular function in a chronic process. In our study, however, CKD could increase the risk of incident HFrEF within 3–12 months even after adjusting for many well-known confounding factors. Some reasons should be noted. The CKD group had more comorbidities like hypertension, DM, CHF, and AF. Pressure and volume overload of the heart caused by high blood pressure and HF may aggravate the deterioration of cardiac function (21). On the other hand, the CKD group was less likely to receive ACEI/ARB. Previous studies have also shown that ACEI/ARB therapy was accompanied with reduced risks of incident HF (2, 22). Moreover, there are some pathophysiologic mechanisms to account for the adverse effect of CKD on incident HFrEF. First, volume overload and renin-angiotensin-aldosterone system activation-mediated cardiac remodeling are known consequences of CKD (23). Moreover, CKD-related abnormalities in calcium transport, fibrinolysis, homocysteine, and systemic inflammation may contribute to the progress of HFrEF as well (26). Besides, the high phosphorus status associated with CKD can promote calcification of cardiac vessels and valves, which further accelerates the process of HFrEF (27).

There are some potential clinical implications from this research. The prevalence of CKD is common in patients undergoing PCI. Therefore, enhancing renal function assessment to screen for CKD is important before PCI, and can remind clinicians of taking measures to protect kidney function, such as formulating hydration measures and reducing the dose of contrast agent (28). In addition, incorporation of CKD in incident HFrEF risk assessment of patients with CAD undergoing PCI can help optimize the selection of high-risk patients who have the potential to obtain the largest advantage of more aggressive cardioprotective prevention treatments (29). Moreover, high-risk patients should be regularly followed up for incident HFrEF in order to detect it early and take renal function protection measures for patients with incident HFrEF such as SGLT2 inhibitors (30). Besides, further studies are needed to explore new strategies to prevent the occurrence of incident HFrEF in patients with CKD undergoing.

## Limitations

Nevertheless, several limitations could be noticed in our study. First, our research was single-center and retrospective, and did not reflect a direct causation. However, our study was large and population-based, and can be a good representative of the population undergoing PCI. Second, there existed a population selection bias when we screened patients, and we excluded patients without follow-up echocardiography. The incidence of HFrEF may be inconsistent among these patients, but we selected a relatively uniform population, and all of them accepted cardiac echocardiography during hospitalization. Third, occurrence of HFrEF is needed to undertake a long-term follow-up, and there was variability in the incidence of HFrEF in the 3–12 months follow-up. However, we mainly evaluated short-term and medium-term incidences, which still could reflect the deterioration of cardiac function. Fourth, the echocardiography data of the patients were collected with a specific time window, but the specific time point was unclear. Finally, our data lacked specific causes of death and information on coronary vessels anatomy, such as number of vessels, entity of coronary stenosis, and target PCI vessels. Therefore, more high-quality studies on the effects of CKD on incident HFrEF following PCI are needed to confirm our findings.

## Conclusion

Our results suggested that 6.9% of the patients with CKD undergoing PCI developed HFrEF, and that the prevalence of incident HFrEF was 2.5 times higher in the CKD group than in the non-CKD group. Moreover, CKD was an independent risk factor for incident HFrEF, which was related to higher all-cause mortality in patients with CAD undergoing PCI. This suggests that early identification of high-risk population and aggressive cardioprotective treatment can help further improve the prognosis of these patients.

## Data Availability Statement

The original contributions presented in the study are included in the article/Supplementary Materials, further inquiries can be directed to the corresponding authors.

## Ethics Statement

The studies involving human participants were reviewed and approved by Guangdong Provincial People's Hospital Ethics Committee. The patients/participants provided their written informed consent to participate in this study.

## Author Contributions

WL, XZ, and SY: research idea and study design. ZM, YZ, ZH, QL, HH, HuL, HW, YX, DG, and HoL: data acquisition. WL: data analysis/interpretation. SL: statistical analysis. YL, SC, and JL: supervision, mentorship, and writing guidance. Each author contributed important intellectual content during manuscript drafting or revision and accepts accountability for the overall study by ensuring that questions on the accuracy or integrity of any portion of the study are appropriately investigated and resolved. All the authors read and approved the final version of the manuscript.

## Funding

This research was funded and supported by Guangdong Provincial science and technology project (2020B1111170011). Guangdong Provincial science and technology project (KJ022021049). The funders had no role in study design, collection and analysis of data, and decision to publish or preparation of the manuscript.

## Conflict of Interest

The authors declare that the research was conducted in the absence of any commercial or financial relationships that could be construed as a potential conflict of interest.

## Publisher's Note

All claims expressed in this article are solely those of the authors and do not necessarily represent those of their affiliated organizations, or those of the publisher, the editors and the reviewers. Any product that may be evaluated in this article, or claim that may be made by its manufacturer, is not guaranteed or endorsed by the publisher.
