# Supplementary material for: Chronic Kidney Disease Increases Risk of Incident HFrEF Following Percutaneous Coronary Intervention
Source: Front Cardiovasc Med. 2022 Apr 1;9:856602. doi: 10.3389/fcvm.2022.856602 (PMC9010558; doi:10.3389/fcvm.2022.856602)
Supplement: Supplementary file 1 [file Table_1.docx]

**Supplemental Table 1**. Baseline characteristics of PCI patients with and without HFrEF.

| **Characteristics** | **Overall** | **Non-HFrEF** | **HFrEF** | **P-Value** | |
| --- | --- | --- | --- | --- | --- |
|  | **(N=2,356)** | **(N=2,273)** | **(N=** **83)** |  |  |
| **Demographic** | | | | | |
| Age, years | 62.5 (10.7) | 62.3 (10.8) | 65.1 (8.6) | 0.023 | |
| Gender, n (%) | 522 (22.2) | 501 (22.0) | 21 (25.3) | 0.570 | |
| **Medical history and Clinical condition** | | | | | |
| AMI, n (%) | 161 (6.8) | 152 (6.7) | 9 (10.8) | 0.211 | |
| VHD, n (%) | 123 (5.2) | 117 (5.2) | 6 (7.2) | 0.558 | |
| AF, n (%) | 62 (2.6) | 57 (2.5) | 5 (6.0) | 0.106 | |
| HT, n (%) | 1,421 (60.3) | 1371 (60.3) | 50 (60.2) | 1.000 | |
| DM, n (%) | 730 (31.0) | 701 (30.9) | 29 (34.9) | 0.503 | |
| CHF, n (%) | 240 (10.2) | 224 (9.9) | 16 (19.3) | 0.009 | |
| **Laboratory examination** | | | | | |
| CK-MB (U/L) | 10.70 [7.30, 16.10] | 10.70 [7.30, 15.90] | 13.30 [7.20, 22.80] | 0.227 | |
| GLU (mmol/L) | 7.46 (3.41) | 7.43(3.39) | 8.45 (4.03) | 0.078 | |
| HbA1c (mmol/L) | 6.62 (1.40) | 6.63 (1.41) | 6.58 (1.13) | 0.817 | |
| LDL-C (mmol/L) | 2.90 (1.02) | 2.90 (1.02) | 2.91 (1.02) | 0.975 | |
| HDL-C (mmol/L) | 0.98 (0.24) | 0.98 (0.24) | 0.99(0.23) | 0.670 | |
| HGB (g/L) | 133.28 (17.25) | 133.36 (17.19) | 131.08 (18.54) | 0.239 | |
| eGFR(ml/min/1.73m2) | 77.27 (24.69) | 77.68 (24.55) | 67.20 (26.01) | | <0.001 |
| NT-Pro-BNP (pg/ml) | 293.80 [78.32, 972.75] | 270.30 [74.35, 870.38] | 1671.00 [955.35, 3211.50] | | <0.001 |
| **Echocardiography** | | | | | |
| LVEF (%) | 60.3 (9.1) | 60.7 (8.9) | 48.6 (7.5) | | <0.001 |
| LVEDD (mm) | 47.9 (5.9) | 47.7 (5.7) | 55.0 (7.0) | | <0.001 |
| LVESD (mm) | 31.3 (6.6) | 31.0 (6.3) | 40.6 (8.0) | | <0.001 |
| MAP (mmHg) | 0.8 (0.8) | 0.8 (0.8) | 0.8 (0.3) | 0.886 | |
| MEP (mmHg) | 0.74 (0.24) | 0.74 (0.24) | 0.75 (0.27) | 0.869 | |
| PWLV (mm) | 10.02 (1.61) | 10.02 (1.61) | 9.85 (1.70) | 0.335 | |
| VS (mm) | 10.62 (1.83) | 10.63 (1.82) | 10.27 (2.13) | 0.078 | |
| **Treatment during hospitalization** | | | | | |
| ACEI/ARB, n (%) | 1,303 (55.4) | 1,254 (55.3) | 49 (55.3) | 0.490 | |
| β-blocker, n (%) | 2,051 (87.2) | 1,974 (87.0) | 77 (94.0) | 0.094 | |
| Statins, n (%) | 2,318 (98.6) | 2,238 (98.6) | 80 (97.6) | 0.739 | |
| CCB, n (%) | 565 (24.0) | 547 (24.1) | 18 (22.0) | 0.751 | |
| **Event** | | | | | |
| Follow up death,  n (%) | 207 (8.8) | 185 (8.1) | 22 (26.5) | | <0.001 |

**Abbreviation:** AMI = acute myocardial infarction; DM= diabetes mellitus; CKD=chronic kidney disease; CHF=congestive heart failure; AF= Atrial fibrillation; VHD= valvular heart disease; PCI= percutaneous coronary intervention; HbA1c=glycosylated hemoglobin; LDL-C=low density lipoprotein cholesterol; HDL-C=High density lipoprotein cholesterol; CK-MB = creatine kinase isoenzyme-MB; GLU= glucose; HGB= hemoglobin; eGFR=estimated glomerular filtration rate; NT-Pro-BNP= N-terminal pro-brain natriuretic peptide; LVEF=left ventricular ejection fraction; LVEDD= left ventricular end-diastolic dimension; LVESD= left ventricular end-systolic dimension; MAP= mean artery pressure; MEP=mean effective pressure; PWLV=posterior wall of left ventricle; VS=ventricular septum; ACEI/ARB=angiotensin-converting enzyme inhibitor/angiotensin receptor blocker; CCB=calcium channel blocker. HFrEF = heart failure with reduced ejection fraction.
