# Supplementary material for: Chronic Kidney Disease Increases Risk of Incident HFrEF Following Percutaneous Coronary Intervention
Source: Front Cardiovasc Med. 2022 Apr 1;9:856602. doi: 10.3389/fcvm.2022.856602 (PMC9010558; doi:10.3389/fcvm.2022.856602)
Supplement: Supplementary file 2 [file Table_2.docx]

**Supplemental Table 2**. Baseline characteristics of included and excluded PCI patients

| **Characteristics** | **Overall** | **Excluded population** | **Enrolled population** | | **P-Value** | |
| --- | --- | --- | --- | --- | --- | --- |
|  | **(N=43,415)** | **(N=41,059)** | **(N=** **2,356)** | |  |  |
| **Demographic** | | | | | | |
| Age, years | 62.8 (10.9) | 62.8 (10.9) | 62.4 (10.7) | | | 0.132 |
| Gender, n (%) | 9,444 (21.8) | 8,922 (21.7) | 522 (22.2) | | | 0.644 |
| **Medical history and Clinical condition** | | | | | | |
| AMI, n (%) | 2,262 (5.2) | 2,669 (6.5) | 161 (6.8) | | | 0.066 |
| VHD, n (%) | 1,415 (3.3) | 1,292 (3.2) | 123 (5.2) | | | <0.001 |
| AF, n (%) | 908 (2.1) | 846 (2.1) | 62 (2.6) | | | 0.070 |
| HT, n (%) | 24,348 (56.2) | 24,676 (60.1) | 1,421 (60.3) | | | 0.056 |
| DM, n (%) | 12,300 (28.4) | 11,570 (28.2) | 730 (31.0) | | | 0.004 |
| CHF, n (%) | 3,954 (9.1) | 3,714 (9.0) | 240 (10.2) | | | 0.069 |
| **Laboratory examination** | | | | | | |
| CK-MB (U/L) | 9.40 [5.90, 14.50] | 9.30 [5.80,  14.40] | 10.70 [7.30,  16.10] | | | <0.001 |
| GLU (mmol/L) | 7.29 (3.40) | 7.29 (3.40) | 7.46 (3.41) | | | 0.070 |
| HbA1c (mmol/L) | 6.60 (1.47) | 6.60 (1.47) | 6.62 (1.40) | | | 0.506 |
| LDL-C (mmol/L) | 2.82 (0.98) | 2.81 (0.97) | 2.90 (1.02) | | | <0.001 |
| HDL-C (mmol/L) | 0.98 (0.25) | 0.98 (0.25) | 0.98 (0.24) | | | 0.908 |
| HGB (g/L) | 133.29 (16.78) | 133.29 (16.75) | 133.28 (17.25) | | | 0.968 |
| eGFR(ml/min/1.73m2) | 77.81 (25.34) | 77.85 (25.38) | 77.27 (24.69) | | | 0.319 |
| NT-Pro-BNP (pg/ml) | 297.35 [81.62, 1139.00] | 297.60 [81.77, 1152.00] | 293.80 [78.32, 972.75] | 0.082 | | |
| **Echocardiography** | | | | | | |
| LVEF (%) | 58.5 (11.9) | 58.3 (12.2) | 60.3 (9.1) | | | <0.001 |
| LVEDD (mm) | 48.43 (6.58) | 48.48 (6.64) | 47.973 (5.92) | | | <0.001 |
| LVESD (mm) | 32.15 (8.16) | 32.23 (8.28) | 31.28 (6.60) | | <0.001 | |
| MAP (mmHg) | 0.83 (1.49) | 0.83 (1.54) | 0.83 (0.75) | | 0.942 | |
| MEP (mmHg) | 0.75 (0.80) | 0.75 (0.83) | 0.74 (0.24) | | 0.885 | |
| PWLV (mm) | 9.91 (1.85) | 9.90 (1.87) | 10.02 (1.61) | | 0.005 | |
| VS (mm) | 10.48 (1.85) | 10.46 (1.85) | 10.62 (1.83) | | <0.001 | |
| **Treatment during hospitalization** | | | | | | |
| ACEI/ARB, n (%) | 23,348 (54.5) | 22,045 (54.5) | 1,303 (55.4) | | 0.374 | |
| β-blocker, n (%) | 36,158 (84.4) | 34,107 (84.3) | 2,051 (87.2) | | <0.001 | |
| Statins, n (%) | 41,801 (97.6) | 39,483 (97.5) | 2,318 (98.6) | | 0.001 | |
| CCB, n (%) | 8,532 (19.9) | 9,731 (23.7) | 565 (24.0) | | 0.533 | |
| **Event** | | | | | | |
| Follow up death,  n (%) | 4,965 (13.34) | 4,758 (13.65) | 207 (8.79) | | <0.001 | |

**Abbreviation:** AMI = acute myocardial infarction; DM= diabetes mellitus; CKD=chronic kidney disease; CHF=congestive heart failure; AF= Atrial fibrillation; VHD= valvular heart disease; PCI= percutaneous coronary intervention; HbA1c=glycosylated hemoglobin; LDL-C=low density lipoprotein cholesterol; HDL-C=High density lipoprotein cholesterol; CK-MB = creatine kinase isoenzyme-MB; GLU= glucose; HGB= hemoglobin; eGFR=estimated glomerular filtration rate; NT-Pro-BNP= N-terminal pro-brain natriuretic peptide; LVEF=left ventricular ejection fraction; LVEDD= left ventricular end-diastolic dimension; LVESD= left ventricular end-systolic dimension; MAP= mean artery pressure; MEP=mean effective pressure; PWLV=posterior wall of left ventricle; VS=ventricular septum; ACEI/ARB=angiotensin-converting enzyme inhibitor/angiotensin receptor blocker; CCB=calcium channel blocker.
